# Malouf Syndrome with Hypergonadotropic Hypogonadism and Cardiomyopathy: Two-Case Report and Literature Review

**DOI:** 10.1155/2014/275710

**Published:** 2014-12-03

**Authors:** Dilek Benk Şilfeler, Atilla Karateke, Raziye Keskin Kurt, Özgür Aldemir, Alper Buğra Nacar, Ali Baloğlu

**Affiliations:** ^1^Department of Obstetrics and Gynecology, Mustafa Kemal University Medical School, Hatay, Turkey; ^2^Clinics of Obstetrics and Gynecology, Hatay Antakya Maternity Hospital, Hatay, Turkey; ^3^Department of Genetics, Mustafa Kemal University Medical School, Hatay, Turkey; ^4^Department of Cardiology, Mustafa Kemal University Medical School, Hatay, Turkey

## Abstract

Malouf syndrome is a very rarely encountered syndrome which was first diagnosed in 1985 upon the examination of two sisters, with findings of hypergonadotropic hypogonadism, dilated cardiomyopathy, blepharoptosis, and broad nasal base. Later on, Narahara diagnosed another sporadic case with the same findings. A survey of relevant literature leads us to three women cases in total. Here we present two cases of Malouf syndrome and literature review.

## 1. Introduction

Malouf syndrome was first diagnosed in 1985 upon the examination of two sisters with findings of hypergonadotropic hypogonadism, dilated cardiomyopathy, blepharoptosis, and broad nasal base [1]. As a consequence of the fact that the parents of these sisters were first-degree cousins, Malouf pointed out that this syndrome was familial. Later on, Narahara diagnosed another sporadic case with the same findings and a detailed characterization was made by autopsy [2]. Here we present two typical cases of Malouf syndrome.

## 2. Case 1

**Figure 1:**
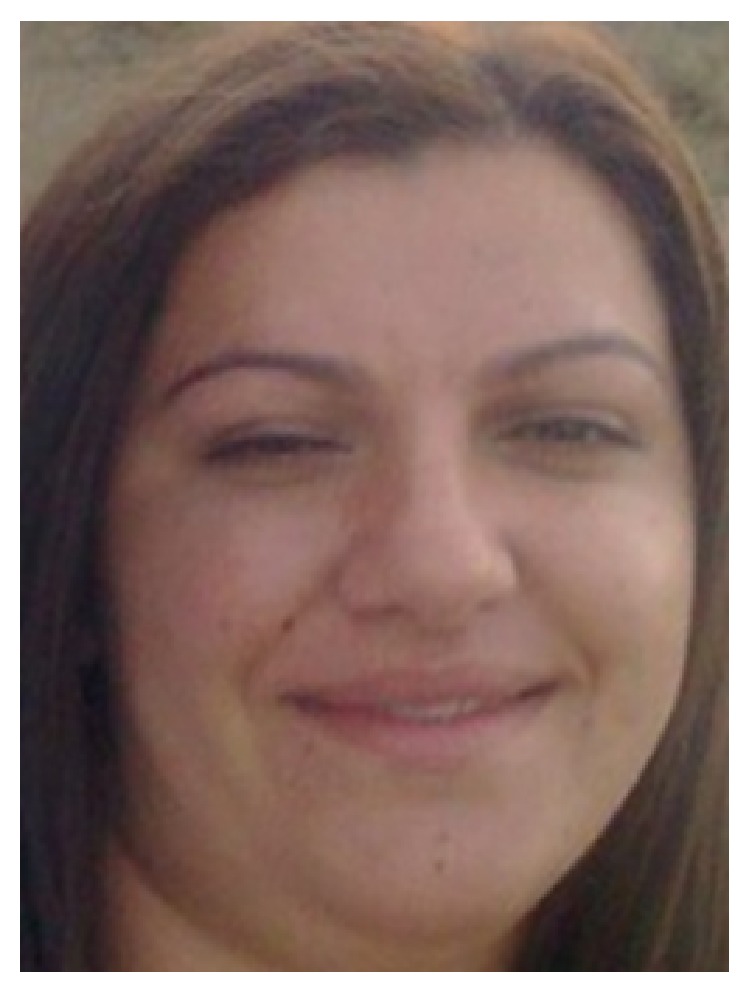
Case 1. Patient's face.

In Figure 1, Twenty-six-year-old women presented to our clinic with a complaint of amenorrhea. She was 165 cm in height and 65 kg in weight. In this patient, a finding of blepharoptosis and a finding of broad nasal base were not very evident (Figure 1). In physical examination her external genital organs were in normal appearance. As the patient was virgin, vaginal examination was not performed. By means of hysterometry, vaginal length was measured as 8 cm. Breast development was concordant with Tanner phase 2. Ultrasonography revealed a hypoplastic uterus and the bilateral ovaries could not be clearly seen. In the magnetic resonance imaging (MRI) examination, it was ascertained that the uterus was smaller than normal dimensions, and bilateral ovaries were not observed. Complete blood count and serum biochemical parameters were found to be normal, and follicle stimulating hormone (FSH) was determined to be 77 IU/L, luteinizing hormone (LH) 25 IU/L, and estradiol 12 mg/dL. These were measured using standard enzymatic methods with a fully automated random access chemiluminescence-enhanced enzyme immunoassay system (Roche Laboratory Systems, Mannheim, Germany). The patient with a chromosome analysis of 46 XX was enabled to menstruate with the support of estradiol and progesteron. After seven years, the patient was consulted to cardiology department because of sudden onset of dyspnea and chest pain. On physical examination she had blood pressure (BP) of 90/65 mmHg, raised jugular venous pressure (JVP), crackles over the lung bases, and a pansystolic murmur at the apex. Besides, her electrocardiogram revealed sinus tachycardia with left bundle brunch block with QRS duration of 160 ms and a normal corrected QT interval. Chest X-ray revealed a prominent cardiomegaly with pulmonary congestion. The patient underwent echocardiography which demonstrated biventricular dilatation with global hypokinesia and moderate mitral valvular regurgitation. Diastolic and systolic diameters of the left ventricle were 69 and 55 mm, respectively. The left ventricular ejection fraction was 20–25%. Pulmonary artery systolic pressure calculated from tricuspid regurgitation was 65 mmHg. After 3-month close follow-up with guideline directed medical therapy, she underwent cardiac resynchronization-defibrillator therapy. From a cardiological viewpoint, the patient was evaluated to be in end-stage and in 2012, she died of cardiomyopathy and cardiac insufficiency.

## 3. Case 2

**Figure 2:**
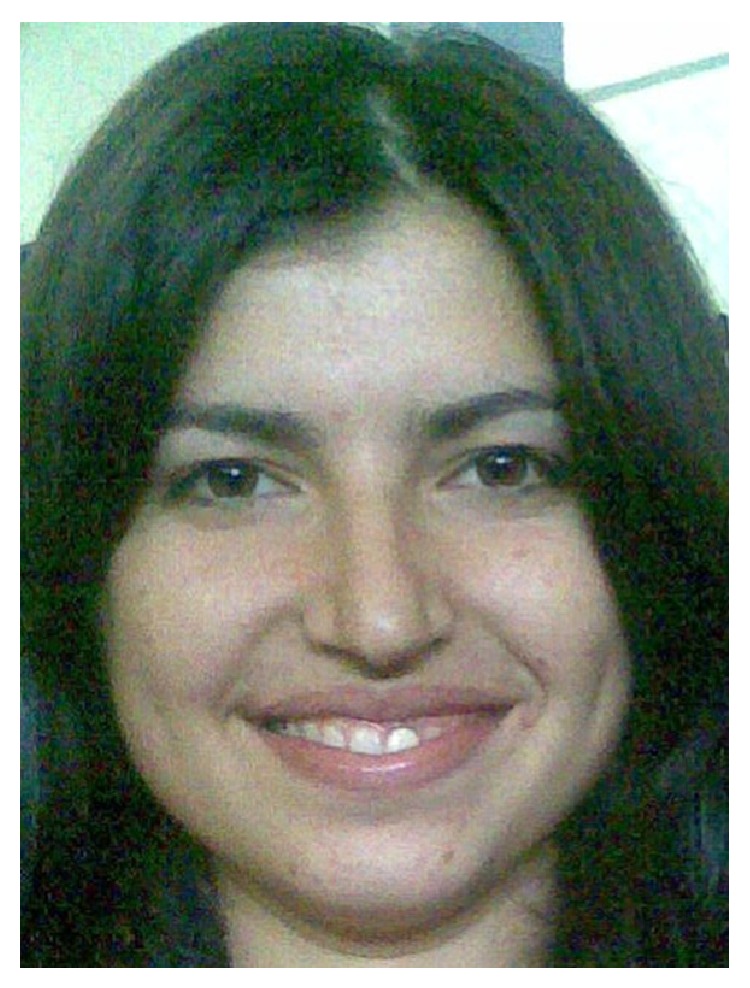
Case 2. Patient's face.

The patient (Figure 2), who was twenty-two years old, 155 cm in height, and 80 kg in weight, applied to the clinic with a complaint of amenorrhea. In this patient, a finding of broad nasal base was not very evident (Figure 2). External genital organs were normal in appearance. As the patient was virgin, no vaginal examination could be performed. By means of hysterometry, vaginal length was measured as 7,5 cm and breast development was concordant with Tanner phase 1. Pelvic ultrasonography revealed that there were no uterus and ovaries. MRI examination indicated that the dimensions of the uterus were extremely small, and bilateral ovaries were absent. FSH value was determined to be 95 IU/L, LH 35 IU/L, and estradiol 10 mg/dL. In the chromosome analysis, the patient was recorded as 46, XX 15q deletion. As the elder sister of the patient had a sudden cardiac death history, cardiological consultation was requested. The electrocardiogram showed sinus tachycardia (heart rate 120/min) with normal axis deviation and nonspecific ST-T wave abnormalities. Chest X-ray showed moderate cardiomegaly with a cardiothoracic ratio of 0.58. On the echocardiogram, left ventricular dilatation was observed with an ejection fraction of 35%. Her echocardiographic findings were consistent with a diagnosis of dilated cardiomyopathy and she followed with on medical therapy.

## 4. Conclusion

Coexistence of gonadal dysgenesis and cardiomyopathy is extremely rare. In relevant literature, there are three women cases who had such a coexistence. The findings of these patients were presented in Table 1. The first two women patients were diagnosed with the same findings as those put forward by Malouf and his colleagues [1]. The last case, on the other hand, was diagnosed in 1992 by Narahara and his colleagues by taking account also of mental retardation and minor skeletal deformities [2]. All patients presented with a common findings of blepharoptosis and a broad nasal base. Dilated cardiomyopathy was observed and cardiovascular symptoms become evident in the second to third decades. Due to cardiac insufficiency mortality occurred in these ages [1, 2]. In all cases, including the two cases of ours, hypergonadotropic hypogonadism was determined.

**Figure 3:**
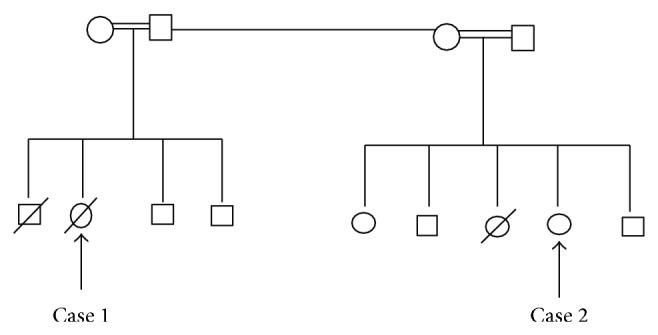
Pedigree.

Malouf and his colleagues diagnosed two sisters and stated that the parents of these sisters were first-degree cousins. In their family story, there is also a mention of the death of two brothers, approximately at the age of eighteen, due to cardiac insufficiency [1]. As remarked in Pedigree, our cases also consist of siblings and cousins (Figure 3). There were also relationships between the parents of the cases. In addition, it was learnt from the detailed medical record that the second case had another sister, who was born in 1998 and died in 2005 due to sudden cardiac arrest. It was reported that the patient complained of dyspnea and fatigue and that she had swellings on her legs which were typical sign and symptoms of dilated cardiomyopathy. From the gynecological medical record, it was ascertained that the case had similar findings to her sister.

It was reported that our first case had a brother, who had died at the age of eight due to a sudden cardiac arrest, and was said to have had dyspnea, fainting, and cardiac rhythm disorder approximately one year before passing away. Thus, the existence of male cases diagnosed with hypergonadotropic hypogonadism, cardiomyopathy, undescended testis, micropenis, and small testis does support the thesis that this situation can be familial and autosomal recessive [3–5].

Unlike our case, there were arachnodactyly, thoracic scoliosis, and mental retardation in the patient diagnosed by Narahara and his colleagues. Besides, there was no relationship between the parents. According to the autopsy reports they performed on their patients, “histologically, the apparent gonadal tissue consisted only of stromal cells and Mullerian tube remnants, with virtually absent oocytes” [2]. Although we could not perform autopsy to our cases, the clinical findings of the patients had parallelism with the clinical findings with Narahara's cases.

Consequently, the coexistence of hypergonadotropic hypogonadism and cardiomyopathy is extremely rare. If such a case is determined, physicians should keep in mind that this situation might be familial. Taking into consideration the high mortality rate of cardiomyopathy, the patient should certainly undergo a cardiac examination in case of hypergonadotropic hypogonadism. Also in case of cardiomyopathy, other family members should be examined.

## Figures and Tables

**Table 1:** 

	Malouf Case 1	Malouf Case 2	Narahara case	Case 1	Case 2
Age at examination	20	26	18	25	22
Karyotype	46XX	46XX	46XX	46XX	46, XX 15q deletion
Mental retardation	−	−	+	−	−
Ptosis	+	+	+	Extremely evident	+
Prominent nasal base	+	+	+	Not so prominent	Not so prominent
Arachnodactyly	−	−	+	−	−
Ovarian dysgenesis	+	+	+	+	+
Cardiomyopathy	Dilated	Dilated	Dilated	Dilated	Dilated
Increased LH level	+	+	+	+	+
Increased FSH level	+	+	+	+	+
